# Nursing Students’ Knowledge and Attitudes Regarding Medical Marijuana: A Descriptive Cross-Sectional Study

**DOI:** 10.3390/ijerph17072492

**Published:** 2020-04-06

**Authors:** Laura Pereira, María Jesús Núñez-Iglesias, Eva María Domínguez-Martís, David López-Ares, Mercedes González-Peteiro, Silvia Novío

**Affiliations:** 1 Galician Public Health Care Service, Lugar Bouza 6, 36164 A Coruña, Spain; laurapereirarosales@gmail.com; 2 School of Nursing, University of Santiago de Compostela, Av. Xoán XXIII, s/n, 15782 Coruña, Spain; mmercedes.gonzalez@usc.es; 3 Galician Public Health Care Service, Health Care Centre of Concepción Arenal, C/ Santiago León de Caracas 12, 15701 A Coruña, Spain; eva.dominguez2@hotmail.com; 4 Galician Public Health Care Service, University Hospital Complex of A Coruña (CHUAC), C/ Xubias de Arriba, 84, 15006 A Coruña, Spain; dlopares@gmail.com

**Keywords:** attitude, knowledge, medical marijuana, nursing

## Abstract

Marijuana use for medical purposes dates back to ancient times. Despite its high therapeutic potential, its adverse effects have raised important legal restrictions. However, this situation in Spain may soon undergo significant changes, without anyone so far having studied the knowledge and/or the level of acceptance of medical marijuana by future healthcare professionals. The aim of the present study was to determine nursing students’ knowledge of and attitudes towards medical marijuana. A cross-sectional design was used. A total of 578 nursing students from the University of Santiago de Compostela (Spain), ≥18 years old and of both sexes, were invited to complete the Spanish version of the questionnaire “Medical Marijuana” between January and May 2019. A total of 364 students decided to participate in the study. More than 75% of the students agreed with the legalization of medical marijuana, although their knowledge and confidence levels regarding efficacy, safety and drug interactions of medical marijuana were low. Nursing students showed a clear lack of knowledge about medical marijuana and thus, in light of possible regulatory changes, it would be necessary to strengthen the training of nurses with respect to medical marijuana in order to make responsible use of it.

## 1. Introduction

The use of marijuana with therapeutic purposes dates back from 2700 BC [1]; however, its psychoactive effects (e.g., confusion, anxiety, etc.) have raised, throughout history, important legal restrictions in terms of investigation and/or prescription, thus potentially restricting its therapeutic use [2].

The term “medical marijuana” refers to a wide variety of preparations; however, the most widely used in clinical practice are cannabinoid-based medicines [3]. Cannabinoids are constituents of cannabis, of which tetrahydrocannabinol (THC) and cannabidiol (CBD) are the most studied so far [4]. While THC, due to its psychoactive properties, has given marijuana a bad name by encouraging its consumption for recreational purposes, CBD lacks such effects [5]. However both cannabinoids are currently commercialized as medicinal products (ex. Sativex, Cesamet, Marinol, etc.) to treat a variety of conditions such as spasticity, chronic pain and others [3,6,7,8,9], despite the potential for side effects [10,11,12,13,14,15,16,17].

The first country to legalize medical marijuana was the United States, followed by Canada [10]; countries in which medical marijuana is acknowledged to have had a long list of indications [3]. However, currently there are many countries in the European Union in which medical marijuana has gained wide acceptance and its use has been authorized [3,10]. For example, marijuana is presently prescribed in Spain for the treatment of spasticity associated with multiple sclerosis [3], and while to date no study has investigated the knowledge and/or the degree of acceptance of medical marijuana by future healthcare professionals, its indications may be expanded in the near future in view of the political initiatives to regulate its use [18]. Advancing understandings of the knowledge and attitudes of healthcare professionals (via an objective evaluation questionnaire) may help to identify ongoing barriers related to marijuana such as knowledge gaps and drug interactions. Besides monitoring and providing care to patients, nursing professionals prepare and administer medications [19], so they should be aware of adverse drug effects. Likewise, and especially as a result of changes in Spanish regulations regarding prescriptions [20], nurses should also have a thorough knowledge of drug indications. Furthermore, nursing professionals take part in developing health behavior programs, which frequently include components that attempt to change attitudes. According to this, the objective of this paper is to determine nursing students’ knowledge about the indications and adverse effects of medical marijuana, as well as to study their attitudes towards the medical and recreational use of marijuana.

## 2. Materials and Methods

### 2.1. Design

A cross-sectional, descriptive, observational study was carried out.

### 2.2. Setting and Participants

All nursing students of the University of Santiago de Compostela (USC, Galicia, Spain), one of the three public universities of Galicia, were invited to participate in the study. The investigation included students enrolled in a nursing degree academic course between 2018 and 2019, of either sex and 18 years or older, who voluntarily agreed to participate. On the contrary, foreign students were excluded from the study.

The size of the study population was 578 at the time of the research (152 of the first year, 133 of the second year, 145 of the third year and 148 of the fourth year). Keeping the expected frequency of all variables at 50%, the desirable sample size using a 95% confidence interval came out to be 309. However, after 15% inflation and rounding, the final desired sample size was determined to be 360.

### 2.3. Translation and Transcultural Adaptation

Translation-backtranslation was the methodology used to make the semantic and cultural adaptation of the questionnaire “Medical Marijuana” [21], following the guidelines of Beaton et al. [22] and Sperber et al. [23].

Two translations of the original version of the questionnaire were made to Spanish by two bilingual people with wide knowledge of pharmacologic therapies. In order to verify the adequacy of the translations, these were revised by the investigator team, and a unified version of the questionnaire was obtained in Spanish (Supplementary Materials, Table S1). Next, the backtranslation process was conducted. Following the same procedure, the unified Spanish version was translated again into English by two people who did not know the original version of the questionnaire. Conceptual and semantic equivalences were analyzed for each one of the items. Finally, a pilot study was carried out with 15 students who did not participate in the final study, in order to evaluate the clarity and ease of understanding of the items. They reported full comprehension of the questions and ease in completing the questionnaire, so no change was carried out.

### 2.4. Data Collection

The information was obtained from the Spanish version (Table S1) of the questionnaire “Medical Marijuana”, developed by Moeller and Woods [21].

The questionnaire consists of 31 questions structured into three sections, which is preceded by eight questions of general information. The first section assesses knowledge of medical marijuana, including therapeutic uses and adverse effects. Only uses [3] and adverse effects [10,11,12,13,14,15,16,17] of cannabinoid-based medicines authorized in European Union countries were included. The second section measures students’ attitudes and opinions regarding medical and recreational use of marijuana and evaluates their confidence levels regarding efficacy, safety and drug interactions of medical marijuana using 23 questions with a five-point Likert scale for each (1 = strongly disagree to 5 = strongly agree). The third section includes seven closed-ended questions related to personal factors that could potentially affect attitudes, opinions and knowledge (sex, age, year of education, marijuana-use history, opinion on whether professors should include information about medical marijuana in classes and information received about medical marijuana during the nursing degree).

In order to assess the reproducibility of the first section of the questionnaire, 15 students who did not participate in the final study filled in the first section of the questionnaire twice at an interval of 15 days between them.

The questionnaires were anonymous and self-completed between January and May of 2019. Students were free to omit any questions they did not want to answer. In order to get high participation levels, two methods were used to distribute the questionnaires: (i) distribution of the questionnaires in person, which were filled in in the break between classes and left on a desk once they were filled in; (ii) mailing of the questionnaires via the application “Google Surveys”.

### 2.5. Ethical and Legal Considerations

The use of the questionnaire “Medical Marijuana” [21] was authorized by the authors of the original instrument, Dr. Moeller and Dr. Woods. The study was performed with the approval of the Faculty of Nursing, University of Santiago de Compostela. Likewise, after explaining the procedure and the objective of the investigation, we obtained the students’ consent that their participation was completely voluntary. Pursuant to the Declaration of Helsinki and Data Protection Act (Organic Law 3/2018), data confidentiality was guaranteed at all times.

### 2.6. Statistical Analysis

The results were presented as number and percentage, mean and standard deviation, or median and confidence interval, as appropriate. Bivariate analysis was performed using ANOVA and Student’s *t*-tests for continuous variables and chi-square tests for categorical variables. Significance between multiple experimental groups was determined using Tukey’s post hoc analysis. In order to compare the answers to the questions of the second section of the questionnaire with the marijuana-use statuses of the four student years, answers were grouped into three categories (disagree = 1 and 2; neutral = 3; agree = 4 and 5). In relation to the psychometric properties of the questionnaire (first section), the test–retest reproducibility was assessed using the kappa statistic. A *p*-value less than 0.05 was considered significant throughout the study. The software SPSS version 24.0 (SPSS Inc., Chicago, IL, USA) was used for the statistical processing of the data.

## 3. Results

A total of 364 students from the Degree of Nursing program of the University of Santiago de Compostela decided to participate in the study with an average response rate of 63%: 88.2% of the first year, 60.9% of the second year, 72.4% of the third year and 29.7% of the fourth year. The remaining students did not answer the questionnaire online or they were absent from class the day the questionnaire was administered. All students who filled in the questionnaires answered all the questions.

Approximately 25% of students stated that the use of medical marijuana was approved in Spain, 30% of participants knew of someone who had used medical marijuana and one-third of students thought that between 10 and 20 countries allowed the use of medical marijuana for authorized indications.

### 3.1. Description of Sample (General Information and Questions Related to Personal Factors)

The demographic characteristics and marijuana-use history of participants are shown in Table 1. The sample was composed primarily of women with a mean age of 20.2 ± 0.6 years. Approximately half of participants reported using marijuana at least once during their lifetimes. One-third of participants thought that marijuana had less adverse effects than alcohol, tobacco or even other drugs.

More than 75% of students supported medical marijuana legalization and 30.8% agreed with its legalization for recreational use. Only a few students (18%) thought that the legalization of recreational marijuana could help increase tax revenue. Regarding its legalization, it is worth noting that students who previously used marijuana were more in favor of the legalization of medical (85.4%) or recreational (44.3%) use compared with students who had never used marijuana (*p* < 0.01).

### 3.2. Knowledge of Medical Marijuana: Indications and Adverse Effects (First Section of the Questionnaire)

Less than 3% of students knew the six potential indications of cannabinoid-based medicines (Table 2), which may have led to the majority of the students (87.6%) who said that professors should include information about medical marijuana in their classes. Cancer (65.4%), multiple sclerosis (39.3%) and muscle spasms (33.2%) were the most common conditions identified by nursing students for the use of cannabinoid-based medicines. On the contrary, the least frequently identified use was HIV/AIDS (human immunodeficiency virus/acquired immunodeficiency syndrome, 3.6%).

Knowledge regarding the indications of cannabinoid-based medicines were dependent on marijuana-use history and the number of years of training in the nursing degree (Table 2). In this sense, it should be noted that nursing students who had previously used marijuana were more knowledgeable than students who reported never having used marijuana regarding two approved indications: cancer and muscle spasms (*p* < 0.05). Likewise, an increase in knowledge occurred from the first- to fourth-year students regarding the use of cannabinoid-based medicines for multiple sclerosis (*p* < 0.001), the only indication approved in Spain.

With respect to the adverse effects of cannabinoid-based medicines (Table 3), paranoia, dizziness and hallucinations were the most common side effects identified by nursing students. Students who had previously used marijuana more often identified nausea as side effect of cannabinoid-based medicines compared with students who reported never having used marijuana (*p* < 0.05); on the contrary, blurred vision and somnolence were most commonly identified by students who had not tried marijuana previously (*p* < 0.05). Likewise, the third year students were the ones who most recognized impaired memory, anxiety, somnolence and nausea as side effects associated with cannabinoid-based medicines.

### 3.3. Attitudes and Opinions About Recreational and Medical Marijuana (Second Section of the Questionnaire)

The most negative attitudes and the worst opinions with regards to medical or recreational marijuana were: “I feel that legalizing medical marijuana would cause more people to use marijuana in non-medical ways”, “I feel that marijuana is a gateway drug” and “I feel that recreational use of marijuana can be detrimental to one’s health”. Furthermore, in general, the most negative attitudes and the worst opinions were found among students who had not previously used marijuana. On the contrary, there were virtually no statistical differences with respect to opinions and/or attitudes towards marijuana among degree years (Table 4).

In relation to level of confidence, all students said they did not feel self-confident answering questions regarding efficacy, safety and potential drug interactions with medical marijuana (*p* > 0.05) (Table 4).

### 3.4. Psychometric Validation

Validation of the questionnaire involved analyzing its reliability (reproducibility). Regarding the test–retest analysis (n = 15), the global kappa was substantial: 0.7414 (95% confidence interval 0.7406–0.7422). On the other hand, content validity was demonstrated, since the questionnaire was based on expert consensus.

## 4. Discussion

To the best of our knowledge, this is the first study that has been carried out to determine the knowledge and opinions of nursing students on medical marijuana with regards to its use and current legislation.

The results of the current study demonstrate that nursing students have a poor knowledge of the potential uses of medical marijuana, as well as the risks associated to it, which corroborates findings from previous studies suggesting that healthcare students need more training on medical marijuana [21,24,25,26]. The students did not know either authorized indications or non-authorized ones. This can be attributed to the constant changes in regulations of medical marijuana use as well as to the wide variations in legislation among European Union member countries [3]. In the current study, only three students stated that they had received some type of information about medical marijuana during their nursing degree; however, more than 85% thought that professors should include information about medical marijuana in their classes. This request could be due to the low level of confidence mentioned by nursing students when answering questions about efficacy, safety and potential drug interactions of marijuana. When we asked where they had received information about marijuana, nursing students answered that it was extracurricular training, in the same way that it has been observed by other authors [26]. The absence of this topic in the nursing degree curriculum, or the curriculums of other healthcare degrees such as medicine [27], could suggest that, despite its acceptance, medical marijuana is still a taboo with many negative connotations. The training of nursing students on medical marijuana could have great implications in Spain, mainly because of two reasons: (i) the only approved indication of cannabinoid-based medicines in Spain is for the treatment of spasticity associated with multiple sclerosis, a neurodegenerative disease that affects 44,000 people in the national territory [28] and whose incidence has increased in the last two decades, turning our country into a high-risk area for multiple sclerosis [29]; and (ii) its legalization for the treatment of other diseases, for which it has already been demonstrated to be effective [3,6,7,8,9], is expected to take place imminently.

On the other hand, we observed that the worst opinions and most negative attitudes about marijuana were concentrated among students who had never consumed it before. This finding is perhaps not surprising, and could be explained by the fact that individual experience with substance use is one of the main factors influencing risk perception and attitudes towards it [30]. As was observed by Paterson and Hammersley [31], substance-using people are more tolerant to smoking, alcohol consumption and illicit drug use than non-users.

Individual experiences with marijuana use also influenced the opinions of the nursing students in relation to its legalization. Students who previously had used marijuana were more in favor of legalization for medical or recreational use, perhaps due to the positive effects identified by the users such as regulation of negative emotions (coping) or avoiding social rejection (conformity) [32,33]. However, the percentage of nursing students that showed support for the legalization of medical marijuana was clearly higher than that in favor of the legalization of recreational marijuana. These results concur with the findings of previous studies carried out among students of other healthcare degrees such as pharmacy [21] or medicine [26]. However, a greater acceptance of the legalization of medical marijuana was registered in our study, which could be attributed to: (i) the coverage that general interest or healthcare newspapers give to this topic, as has already been suggested by D’Amico et al. [34]; or (ii) that Spain is a country with a high cannabis consumption [35], which could have influenced the students’ attitudes toward its legalization [21,26,36].

Over the years, healthcare students have demonstrated to be more receptive to the legalization of marijuana, especially for medical purposes ([21,24,36,37] and current study). Long past are the times when only 16% of pharmacy or medicine students declare they were in favor of the legalization of medical marijuana [24]. It has been suggested that more favorable attitudes towards its use could be attributed to changes in legalization [38], to its higher cultural acceptance [38], to individuals’ own marijuana-use histories (regardless of purpose) [21] or even to a decrease in the perception of risk of marijuana use [39]. The two last explanations were apparent in the current study, since legalization was better received among previous users of marijuana, who also considered it to have less adverse effects than alcohol (*p* < 0.001), tobacco (*p* = 0.001) or even other drugs, although in the latter case statistical significance was not reached (*p* = 0.217).

The profile of healthcare students could influence their opinions or attitudes towards marijuana. The future nurses had more positive opinions and attitudes towards the use of marijuana than the future doctors, with ~50% of medical students indicating that they would never recommend marijuana as a medical treatment [27]. These discrepancies could respond to differences in timing, as mentioned in the previous paragraph, or they could be attributed to medical students having better training in medical marijuana; however, this does not seem to be the case [26]. This could also be a reflection of the responsibility that comes with prescribing marijuana, as well as the side effects that are associated with it.

The results of this study should be interpreted with caution, given the limitations of the study. In particular, the investigation only was carried out with nursing students of one university in which medical marijuana is not included in the curriculum of the degree. Although the results obtained could be extrapolated to the remaining Spanish faculties of nursing, these may differ a lot with respect to countries where the use of medical marijuana is more widespread, such s the Czech Republic or Germany. On the other hand, bearing in mind the sensitive nature of asking about previous marijuana use, information bias was probably present, indicating that observed prevalence may have been underestimated.

## 5. Conclusions

Nursing students showed a clear lack of knowledge about the potential uses of medical marijuana and the risks associated with it; therefore, nurses as well as other healthcare professionals may need to take an education course about marijuana as a prerequisite to their clinical practice in the same manner currently required in other countries.

## Figures and Tables

**Table 1:** Demographic and other characteristics of nursing students. Statistical significance (*p* < 0.05) was determined by chi-square test.

	Marijuana Status				Program				
	All Students N = 364 n (%)	Previous Users N = 178 (48.9%) n (%)	Never Used N = 186 (51.1%) n (%)	*p*	First Year N = 134 (88.2%) n (%)	Second Year N = 81 (60.9%) n (%)	Third Year N = 105 (72.4%) n (%)	Fourth Year N = 44 (29.7%) n (%)	*p*
**Sex**									
Female	306 (84.1)	142 (79.8)	163 (87.6)	0.052	111 (82.8)	70 (86.4)	85 (81.0)	39 (88.6)	0.596
Male	58 (15.9)	36 (20.2)	23 (12.4)		23 (17.2)	11 (13.6)	20 (19.0)	5 (11.4)	
**Program**									
First year	134 (36.8)	55 (30.9)	79 (42.5)	0.14	-	-	-	-	
Second year	81 (22.3)	42 (23.6)	39 (21.0)		-	-	-	-	
Third year	105 (28.8)	58 (32.6)	47 (25.3)		-	-	-	-	
Fourth year	44 (12.1)	23 (12.9)	21 (11.3)		-	-	-	-	
**Legalization**									
For medical marijuana	287 (78.8)	152 (85.4)	135 (72.6)	0.003	108 (80.6)	69 (85.2)	77 (73.3)	33 (75.0)	0.212
For recreational marijuana	112 (30.8)	79 (44.3)	33 (17.7)	<0.001	40 (29.9)	33 (40.7)	26 (24.8)	13 (29.5)	0.13

**Table 2:** Uses of cannabinoid-based medicines authorized in European Union countries. Students were asked about which of the indications suggested were indications of cannabinoid-based medicines. Statistical significance (*p* < 0.05) was determined by chi-square test. Abbreviations: AIDS, acquired immunodeficiency syndrome; HIV, human immunodeficiency virus.

	Marijuana Status				Program				
	All Students N = 364 n (%)	Previous Users N = 178 (48.9%) n (%)	Never Used N = 186 (51.1%) n (%)	*p*	First Year N = 134 (88.2%) n (%)	Second Year N = 81 (60.9%) n (%)	Third Year N = 105 (72.4%) n (%)	Fourth Year N = 44 (29.7%) n (%)	*p*
**Authorized indications**									
Cancer	238 (65.4)	131 (73.6)	107 (57.5)	0.001	80 (59.7)	54 (66.7)	71 (67.6)	33 (75.0)	0.261
Multiple sclerosis	143 (39.3)	73 (41.0)	70 (37.6)	0.51	35 (26.1)	31 (38.3)	54 (51.4)	23 (52.3)	<0.001
Muscle spasm	121 (33.2)	69 (38.8)	52 (28.0)	0.029	44 (32.8)	23 (28.4)	41 (39.0)	13 (29.5)	0.435
Nausea/ Vomiting	35 (9.6)	20 (11.2)	15 (8.0)	0.305	14 (10.4)	10 (12.3)	7 (6.7)	4 (9.1)	0.601
Stimulation of appetite	26 (7.1)	16 (9.0)	10 (5.4)	0.181	13 (9.7)	3 (3.7)	9 (8.6)	1 (2.3)	0.198
HIV/ AIDS	13 (3.6)	3 (1.7)	10 (5.4)	0.058	9 (6.7)	0 (0)	2 (1.9)	2 (4.5)	0.05
**Non-authorized indications**									
Migraines	191 (52.5)	108 (60.7)	83 (44.6)	0.002	81 (60.4)	43 (53.1)	46 (43.8)	21 (47.7)	0.072
Amyotrophic lateral sclerosis	162 (44.5)	85 (47.8)	77 (41.4)	0.223	46 (34.3)	31 (38.3)	55 (52.4)	30 (68.2)	<0.001
Parkinson’s disease	105 (28.8)	59 (33.1)	46 (24.7)	0.076	37 (27.6)	25 (30.9)	34 (32.4)	9 (20.5)	0.492
Epilepsy	84 (23.1)	39 (21.9)	45 (24.2)	0.605	32 (23.9)	23 (28.4)	24 (22.9)	5 (11.4)	0.192
Cystic fibrosis	65 (17.9)	29 (16.3)	36 (19.4)	0.446	16 (11.9)	10 (12.3)	26 (24.8)	13 (29.5)	0.006
Schizophrenia	51 (14.0)	27 (15.2)	24 (12.9)	0.534	16 (11.9)	19 (23.5)	13 (12.4)	3 (6.8)	0.035
Vertigo	41 (11.3)	25 (14.0)	16 (8.6)	0.101	19 (14.2)	9 (11.1)	9 (8.6)	4 (9.1)	0.55
Alzheimer’s disease	41 (11.3)	24 (13.5)	17 (9.1)	0.19	14 (10.4)	14 (17.3)	7 (6.7)	6 (13.6)	0.139
Crohn’s disease	30 (8.2)	9 (5.1)	21 (11.3)	0.031	16 (11.9)	2 (2.5)	7 (6.7)	5 (11.4)	0.075
Glaucoma	28 (7.7)	18 (10.1)	10 (5.4)	0.09	7 (5.2)	3 (3.7)	13 (12.4)	5 (11.4)	0.07
Huntington’s disease	23 (6.3)	8 (4.5)	15 (8.1)	0.162	10 (7.5)	3 (3.7)	5 (4.8)	5 (11.4)	0.314
Gilles de la Tourette syndrome	12 (3.3)	9 (5.1)	3 (1.6)	0.066	0 (0)	3 (3.7)	7 (6.7)	2 (4.5)	0.036
Hepatitis C	3 (0.8)	1 (0.6)	2 (1.1)	0.588	3 (2.2)	0 (0)	0 (0)	0 (0)	0.158

**Table 3:** Adverse effects of cannabinoid-based medicines authorized in European Union countries. Students were asked about which of the adverse effects indicated were adverse effects associated with cannabinoid-based medicines. Statistical significance (*p* < 0.05) was determined by chi-square test.

	Marijuana Status				Program				
	All Students N = 364 n (%)	Previous Users N = 178 (48.9%) n (%)	Never Used N = 186 (51.1%) n (%)	*p*	First Year N = 134 (88.2%) n (%)	Second Year N = 81 (60.9%) n (%)	Third Year N = 105 (72.4%) n (%)	Fourth year N = 44 (29.7%) n (%)	*p*
**Adverse effects of cannabinoid-based medicines**									
**Yes**									
Paranoia	293 (80.5)	150 (84.3)	143 (76.9)	0.075	110 (82.1)	66 (81.5)	85 (81.0)	32 (72.7)	0.578
Dizziness	278 (76.4)	142 (79.8)	136 (73.1)	0.135	102 (76.1)	66 (81.5)	81 (77.1)	29 (65.9)	0.275
Hallucinations	270 (74.2)	124 (69.7)	146 (78.5)	0.054	93 (69.4)	57 (70.4)	86 (81.9)	34 (77.3)	0.127
Blurred vision	243 (66.8)	108 (60.7)	135 (72.6)	0.016	82 (61.2)	64 (79.0)	68 (64.8)	29 (65.9)	0.056
Impaired memory	219 (60.2)	110 (61.8)	109 (58.6)	0.534	78 (58.2)	40 (49.4)	76 (72.4)	25 (56.8)	0.012
Anxiety	193 (53.0)	97 (54.5)	96 (51.6)	0.582	62 (46.2)	43 (53.1)	68 (64.8)	20 (45.5)	0.026
Tachycardia	191 (52.5)	97 (54.5)	94 (50.5)	0.450	69 (51.5)	49 (60.5)	54 (51.4)	19 (43.2)	0.295
Somnolence	173 (47.5)	72 (40.4)	101 (54.3)	0.008	53 (39.6)	35 (43.2)	65 (61.9)	20 (45.5)	0.005
Depression	171 (46.9)	86 (48.3)	85 (45.7)	0.617	57 (42.5)	37 (45.7)	57 (54.3)	20 (45.5)	0.333
Nausea	171 (46.9)	94 (52.8)	77 (41.4)	0.029	59 (44.0)	43 (53.1)	56 (53.3)	13 (29.5)	0.033
Birth defects	50 (13.7)	21 (11.8)	29 (15.6)	0.293	15 (11.2)	13 (16.0)	19 (18.1)	3 (6.8)	0.207
**No**									
Worsening asthma	177 (48.6)	93 (52.2)	84 (45.2)	0.176	52 (38.8)	41 (50.6)	69 (65.7)	15 (34.1)	<0.001
Lung cancer	137 (37.6)	86 (48.3)	51 (27.4)	<0.001	46 (34.3)	24 (29.6)	55 (52.4)	12 (27.3)	0.002
Seizures	109 (29.9)	53 (29.8)	56 (30.1)	0.945	39 (29.1)	24 (29.6)	35 (33.3)	11 (25.0)	0.768
Stroke	97 (26.6)	48 (27.0)	49 (26.3)	0.893	41 (30.6)	15 (18.5)	32 (30.5)	9 (20.5)	0.141
Constipation	42 (11.5)	20 (11.2)	22 (11.8)	0.860	11 (8.2)	6 (7.4)	16 (15.2)	9 (20.5)	0.054
Anemia	30 (8.2)	15 (8.4)	15 (8.1)	0.900	7 (5.2)	7 (8.6)	8 (7.6)	8 (18.2)	0.059
Haemorrhage	10 (2.7)	3 (1.7)	7 (3.8)	0.225	3 (2.2)	5 (6.2)	1 (1.0)	1 (2.3)	0.173
Cataract	7 (1.9)	5 (2.8)	2 (1.1)	0.229	3 (2.2)	0	3 (2.9)	1 (2.3)	0.537
Diabetes	2 (0.5)	2 (1.1)	0	0.147	0	1 (1.2)	1 (1.0)	0	0.574

**Table 4:** Attitudes and opinions about recreational and medical marijuana. Answers were expressed on a five-point Likert scale (1 = strongly disagree to 5 = strongly agree). In order to compare the answers to the questions about opinions and attitudes regarding marijuana-use statuses for the four student years, answers were grouped into three categories (disagree = 1 and 2; neutral = 3; agree = 4 and 5). Statistical significance (*p* < 0.05) was determined by ANOVA or Student’s *t*-tests.

	Marijuana Status				Program				
	All Students N = 364 Median (CI)	Previous Users N = 178 (48.9%) Median (CI)	Never Used N = 186(51.1%) Median (CI)	*p*	First Year N = 134 (88.2%) Median (CI)	Second Year N = 81 (60.9%) Median (CI)	Third year N = 105 (72.4%) Median (CI)	Fourth year N = 44 (29.7%) Median (CI)	*p*
*A. Medical use of marijuana*									
In my opinion, marijuana should be legalized for medicinal uses	3.9 (3.8–4)	4.1 (3.9–4.2)	3.6 (3.5–3.8)	<0.001	3.8 (3.6–4)	4 (3.8–4.2)	3.8 (3.6–4)	3.9 (3.5–4.2)	0.592
In my opinion, all clinicians with prescribing rights (e.g., advanced nurse practitioners, physician assistants) should be able to prescribe medical marijuana	3.2 (3.1–3.4)	3.4 (3.2–3.5)	3.1 (3–3.3)	0.054	3.3 (3.1–3.5)	3.4 (3.2–3.7)	3.1 (2.8–3.3)	3.1 (2.7–3.3)	0.284
I feel that marijuana is safe when used responsibly for medical use	3.9 (3.8–4)	4.1 (4–4.2)	3.8 (3.6–3.9)	0.066	4 (3.8–4.1)	4.1 (4–4.3)	3.8 (3.6–4)	3.9 (3.7–4.2)	0.059
I feel that marijuana can be detrimental to one’s health for medical use	2.9 (2.8–3)	2.8 (2.7–2.9)	3.1 (3–3.2)	0.011	3.0 (2.8–3.1)	2.8 (2.7–3)	3 (2.8–3.2)	2.9 (2.6–3.2)	0.502
I feel that medical marijuana is often abused	2.6 (2.5–2.7)	2.3 (2.1–2.4)	2.8 (2.7–3)	<0.001	2.6 (2.5–2.8)	2.5 (2.4–2.7)	2.6 (2.4–2.8)	2.2 (2–2.5)	0.456
I feel that legalizing medical marijuana would cause crimes rates to increase	2.6 (2.5–2.7)	2.3 (2.2–2.5)	2.9 (2.7–3.1)	<0.001	2.6 (2.5–2.8)	2.6 (2.3–2.8)	2.6 (2.3–2.8)	2.8 (2.5–3)	0.25
I feel that legalizing medical marijuana would cause more people to use marijuana in non-medical ways.	3.5 (3.4–3.6)	3.3 (3.1–3.4)	3.7 (3.5–3.8)	0.01	3.5 (3.3–3.7)	3.5 (3.3–3.7)	3.6 (3.3–3.8)	3.4 (3.2–3.8)	0.553
I feel that legalizing medical marijuana will hurt the “war on drugs” effort	3 (2.9–3.1)	2.8 (2.7–3)	3.2 (3.1–3.4)	<0.001	3.1 (2.9–3.3)	3 (2.8–3.3)	3 (2.8–3.3)	2.9 (2.6–3.2)	0.936
I feel that medical marijuana is safe to use with prescription medications	3.6 (3.5–3.7)	3.7 (3.6–3.8)	3.5 (3.4–3.7)	0.052	3.7 (3.5–3.8)	3.8 (3.6–4)	3.4 (3.2–3.6)	3.5 (3.3–3.7)	0.063
I feel that medical marijuana is safe to use with non-prescription medications	1.9 (1.8–2)	2 (1.9–2.1)	1.7 (1.6–1.8)	0.016	1.8 (1.7–2)	2 (1.8–2.2)	1.8 (1.6–1.9)	1.9 (1.6–2.2)	0.07
I feel that medical marijuana has been adequately studied by scientists	2.6 (2.5–2.7)	2.6 (2.5–2.7)	2.6 (2.5–2.7)	0.56	2.8 (2.7–2.9)	2.6 (2.4–2.7)	2.4 (2.3–2.6)	2.5 (2.3–2.7)	0.051
I feel that the majority of people who support the legalization of medical marijuana are drug abusers	1.8 (1.7–1.9)	1.7 (1.6–1.9)	1.9 (1.8–2.1)	0.095	1.9 (1.8–2.1)	1.7 (1.5–1.9)	1.8 (1.6–1.9)	1.8 (1.5–2.2)	0.116
If I had to make a decision today about legalization of medical marijuana, I would be in favor of doctor-prescribed medical marijuana	3.8 (3.7–3.9)	4 (3.8–4.1)	3.5 (3.4–3.7)	<0.001	3.7 (3.6–3.9)	3.9 (3.7–4.1)	3.7 (3.5–3.9)	3.8 (3.5–4.1)	0.32
I feel that our government has adequate resources to regulate the use of medical marijuana	3.1 (3–3.2)	3.3 (3.2–3.4)	3 (2.8–3.1)	<0.001	3 (2.9–3.2)	3.3 (3–3.5)	3.1 (2.8–3.3)	3.1 (2.7–3.5)	0.058
*B. Recreational use of marijuana*									
In my opinion, marijuana should be legalized for the general population	2.3 (2.2–2.4)	2.6 (2.4–2.8)	2 (1.8–2.1)	<0.001	2.2 (2–2.4)	2.5 (2.3–2.8)	2.2 (2–2.5)	2.3 (1.8–2.5)	0.701
I feel that marijuana is a gateway drug	3.5 (3.3–3.6)	3.4 (3.2–3.6)	3.5 (3.4–3.7)	0.06	3.5 (3.3–3.6)	3.3 (3.1–3.5)	3.6 (3.4–3.8)	3.5 (3.1–3.8)	0.746
I feel that marijuana is safe when used responsibly for recreational use.	1.9 (1.8–2)	2.1 (2–2.2)	1.8 (1.7–1.9)	0.008	1.9 (1.7–2)	2.1 (2–2.4)	2 (1.8–2.2)	1.7 (1.4–2)	0.544
I feel that recreational use of marijuana can be detrimental to one’s health	4.2 (4.1–4.3)	4.1 (3.9–4.2)	4.3 (4.1–4.4)	0.2	4.3 (4.2–4.4)	3.9 (3.7–4.1)	4.2 (4–4.3)	4.1 (3.8–4.5)	0.202
I feel that legalizing marijuana for any use would cause crime rates to increase.	3 (2.9–3.1)	2.8 (2.6–2.9)	3.2 (3.1–3.4)	0.003	3.1 (2.9–3.3)	2.9 (2.6–3.1)	2.9 (2.7–3.1)	3.2 (2.8–3.6)	0.112
*C. Confidence levels*									
I consider myself knowledgeable on the subject of medical marijuana	1.5 (1.4–1.6)	1.6 (1.5–1.7)	1.4 (1.3–1.5)	0.15	1.4 (1.3–1.6)	1.6 (1.5–1.8)	1.5 (1.4–1.6)	1.5 (1.2–1.8)	0.14
I feel comfortable answering questions from my patients about the efficacy of medical marijuana	1.5 (1.4–1.6)	1.6 (1.5–1.7)	1.5 (1.4–1.6)	0.857	1.5 (1.4–1.6)	1.6 (1.4–1.8)	1.4 (1.3–1.6)	1.6 (1.3–1.9)	0.405
I feel comfortable answering questions from my patients about the safety of medical marijuana	1.5 (1.4–1.6)	1.5 (1.4–1.6)	1.4 (1.3–1.5)	0.952	1.5 (1.4–1.7)	1.5 (1.4–1.7)	1.4 (1.3–1.5)	1.4 (1.1–1.7)	0.676
I feel comfortable answering questions from my patients about drug interactions with medical marijuana	1.5 (1.4–1.6)	1.6 (1.5–1.7)	1.4 (1.3–1.6)	0.769	1.6 (1.4–1.7)	1.5 (1.4–1.7)	1.5 (1.4–1.7)	1.5 (1.2–1.8)	0.809

Abbreviations: CI, confidence interval.

## Supplementary Materials

The following are available online at https://www.mdpi.com/1660-4601/17/7/2492/s1, Table S1: Spanish version of the questionnaire Medical marijuana.
