# Supplementary material for: Nursing Students’ Knowledge and Attitudes Regarding Medical Marijuana: A Descriptive Cross-Sectional Study
Source: Int J Environ Res Public Health. 2020 Apr 6;17(7):2492. doi: 10.3390/ijerph17072492 (PMC7177422; doi:10.3390/ijerph17072492)
Supplement: Supplementary file 1 [file ijerph-17-02492-s001.pdf]

Table S1. Spanish version of the questionnaire Medical marijuana. Adapted from Moeller and Woods [21].

Información general:

1. ¿Está aprobado el uso de la marihuana medicinal en España?

   □ Sí □ No □ No sabe/No contesta

2. ¿Piensa que todos los países deberían aprobar el uso de la marihuana medicinal?

   □ Sí □ No □ No sabe/No contesta

3. ¿Cree que la marihuana debería ser legalizada para uso recreativo?

   □ Sí □ No □ No sabe/No contesta

Si ha respondido NO a la cuestión nº 3 siga en la cuestión nº 5.

4. ¿Piensa que la principal razón por la cual la marihuana debería ser legalizada para uso recreativo es para ayudar al incremento de los ingresos fiscales?

   □ Sí □ No □ No sabe/No contesta

5. ¿Piensa que la marihuana tiene más efectos negativos que el alcohol?

   □ Sí □ No □ No sabe/No contesta

6. ¿Piensa que la marihuana tiene más efectos negativos que el tabaco?

   □ Sí □ No □ No sabe/No contesta

7. ¿Piensa que la marihuana tiene más efectos negativos que los medicamentos con prescripción médica?

   □ Sí □ No □ No sabe/No contesta

8. ¿Cuántos países cree que permiten el uso de marihuana bajo prescripción médica?
   □ <10 □ 10-20 □ 21-30 □ 31-40 □ 41-50 □ No sabe/No contesta

PRIMERA PARTE: **Conocimientos sobre la marihuana medicinal.**

La marihuana medicinal incluye una amplia variedad de preparaciones; sin embargo, las más ampliamente utilizadas en la práctica clínica son las medicinas basadas en canabinoides (MBC), cuya autorización varía según los países.

A. Usos médicos

De la lista que se incluye a continuación, por favor seleccione la/s enfermedad/es para la/s cuál/es cree que está indicado el uso de MBC en al menos un país de la Unión Europea.

□ Enfermedad del Alzheimer
□ Esclerosis lateral amiotrófica (ELA)
□ Esclerosis múltiple
□ Espasmos musculares

□ Cáncer
□ Enfermedad de Crohn
□ Fibrosis quística
□ Epilepsia
□ Glaucoma
□ Hepatitis C
□ Enfermedad de Huntington
□ VIH/SIDA
□ Migrañas
□ Náuseas/Vómitos
□ Parkinson
□ Esquizofrenia
□ Síndrome de Tourette
□ Vértigo
□ Estimulación del apetito
□ Ninguna de las anteriores
□ No sabe/no contesta

B. Efectos adversos

De la lista que se incluye a continuación, por favor seleccione el/los efecto/s adverso/s que se asocian con las MBC.

□ Anemia
□ Ansiedad
□ Visión borrosa
□ Cataratas
□ Estreñimiento
□ Depresión
□ Diabetes
□ Mareo
□ Alucinaciones
□ Náuseas
□ Pérdidas de memoria
□ Hemorragia
□ Somnolencia
□ Cáncer de pulmón
□ Defectos congénitos
□ Paranoia
□ Convulsiones
□ Accidente cerebro vascular
□ Taquicardia
□ Empeoramiento del asma
□ Ninguna de las anteriores
□ No sabe/No contesta

SEGUNDA PARTE: **Actitudes y opiniones sobre la marihuana**

Instrucciones: En una escala de 1 (totalmente en desacuerdo) a 5 (totalmente de acuerdo), por favor, indique su grado de conformidad con cada una de las siguientes afirmaciones rodeando la respuesta que le parezca más oportuna.

C. Marihuana de uso medicinal

	Totalmente en desacuerdo	En desacuerdo	Ni acuerdo ni desacuerdo	De acuerdo	Totalmente de acuerdo
1. En mi opinión, la marihuana debería ser legalizada para uso médico.	1	2	3	4	5
2. En mi opinión, todos los profesionales con derecho para prescribir fármacos (enfermeros, psiquiatras…)	1	2	3	4	5

deberían poder prescribir marihuana medicinal.					
3. Pienso que la marihuana medicinal es segura cuando es usada responsablemente con fines terapéuticos.	1	2	3	4	5
4. Pienso que la marihuana medicinal puede ser perjudicial para la salud.	1	2	3	4	5
5. Pienso que se abusa de la marihuana medicinal.	1	2	3	4	5
6. Pienso que la legalización de la marihuana medicinal podría causar un aumento de los índices de delincuencia.	1	2	3	4	5
7. Pienso que la legalización de la marihuana medicinal causaría un mayor consumo de esta sustancia en situaciones no médicas.	1	2	3	4	5
8. Pienso que la legalización de la marihuana medicinal será contraproducente para la "guerra contra las drogas"	1	2	3	4	5
9. Pienso que el uso de marihuana medicinal es seguro bajo prescripción médica.	1	2	3	4	5
10. Pienso que el uso de marihuana medicinal es seguro sin necesidad de prescripción médica.	1	2	3	4	5
11. Pienso que la marihuana medicinal ha sido adecuadamente investigada.	1	2	3	4	5
12. Pienso que la mayoría de la gente que apoya la legalización de la marihuana medicinal son drogadictos.	1	2	3	4	5
13. Si tuviese que tomar hoy una decisión sobre la legalización de la marihuana medicinal, sería a favor de su prescripción médica.	1	2	3	4	5

14. Considero que nuestro gobierno posee los recursos necesarios para regular el uso de la marihuana medicinal.	1	2	3	4	5

D. Marihuana de uso recreativo.

	Totalmente en desacuerdo	En desacuerdo	Ni acuerdo ni desacuerdo	De acuerdo	Totalmente de acuerdo
15. En mi opinión, la marihuana debería ser legalizada para toda la población.	1	2	3	4	5
16. Pienso que la marihuana es una droga de inicio.	1	2	3	4	5
17. Pienso que la marihuana es segura cuando se usa de manera responsable con fines recreativos.	1	2	3	4	5
18. Pienso que la marihuana de uso recreativo puede ser perjudicial para la salud.	1	2	3	4	5
19. Creo que la legalización de la marihuana para cualquier uso causaría un aumento de los índices de delincuencia.	1	2	3	4	5

E. Grado de confianza.

	Totalmente en desacuerdo	En desacuerdo	Ni acuerdo ni desacuerdo	De acuerdo	Totalmente de acuerdo
20. Considero que soy un/a experto/a en el tema de la marihuana medicinal.	1	2	3	4	5
21. Me siento seguro/a respondiendo a los pacientes preguntas sobre la eficacia de la marihuana medicinal.	1	2	3	4	5
22. Me siento seguro/a respondiendo a los pacientes preguntas sobre la interacción farmacológica de la marihuana medicinal.	1	2	3	4	5
23. Me siento seguro/a respondiendo a los	1	2	3	4	5

pacientes preguntas sobre la seguridad de la marihuana medicinal.					

TERCERA PARTE: **Cuestiones personales (por favor, seleccione la respuesta más apropiada)**

1. ¿Alguna vez ha consumido marihuana (de forma médica o no médica)?

   □ Sí □ No □ No sabe/No contesta

2. ¿Conoce a alguien que haya consumido marihuana por motivos médicos?

   □ Sí □ No □ No sabe/No contesta

3. ¿Piensa que los profesores deberían incluir información sobre marihuana medicinal en sus clases?

   □ Sí □ No □ No sabe/No contesta

4. Género:

   □ Masculino □ Femenino

5. Edad:

   □ 18 □ 19 □ 20 □ 21 □ 22 □ Otra: ____

6. Seleccione el año de grado que está cursando:

   □ 1º □ 2º □ 3º □ 4º □ No sabe/No contesta

7. Durante su formación, ¿ha recibido alguna educación específica sobre la marihuana medicinal?

   □ Sí □ No □ No estoy seguro/a

Si la respuesta es "Sí", por favor indique brevemente dónde recibió estas lecciones sobre marihuana medicinal:

